# Shared neural representations of tactile roughness intensities by somatosensation and touch observation using an associative learning method

**DOI:** 10.1038/s41598-018-37378-w

**Published:** 2019-01-11

**Authors:** Junsuk Kim, Isabelle Bülthoff, Sung-Phil Kim, Heinrich H. Bülthoff

**Affiliations:** 1 Department of Human Perception, Cognition and Action, Max Planck Institute for Biological Cybernetics, Tübingen, 72076 Germany; 2 Center for Neuroscience Imaging Research, Institute for Basic Science (IBS), Suwon, 16419 Republic of Korea; 3 Department of Biomedical Engineering, Sungkyunkwan University, Suwon, 16419 Republic of Korea; 4 Department of Human Factors Engineering, Ulsan National Institute of Science and Technology, Ulsan, 44919 Republic of Korea

## Abstract

Previous human fMRI studies have reported activation of somatosensory areas not only during actual touch, but also during touch observation. However, it has remained unclear how the brain encodes visually evoked tactile intensities. Using an associative learning method, we investigated neural representations of roughness intensities evoked by (a) tactile explorations and (b) visual observation of tactile explorations. Moreover, we explored (c) modality-independent neural representations of roughness intensities using a cross-modal classification method. Case (a) showed significant decoding performance in the anterior cingulate cortex (ACC) and the supramarginal gyrus (SMG), while in the case (b), the bilateral posterior parietal cortices, the inferior occipital gyrus, and the primary motor cortex were identified. Case (c) observed shared neural activity patterns in the bilateral insula, the SMG, and the ACC. Interestingly, the insular cortices were identified only from the cross-modal classification, suggesting their potential role in modality-independent tactile processing. We further examined correlations of confusion patterns between behavioral and neural similarity matrices for each region. Significant correlations were found solely in the SMG, reflecting a close relationship between neural activities of SMG and roughness intensity perception. The present findings may deepen our understanding of the brain mechanisms underlying intensity perception of tactile roughness.

## Introduction

Visual assessment of a surface texture prior to actually touching is crucial when interacting with objects^1^. In particular, perceiving the intensity of surface characteristics is of great importance to refine planning of grasping movement. For example, it is necessary to perceive precise roughness intensity before touching sandpaper to avoid skin abrasions or exerting grasping forces to avoid dropping a slippery object. A number of human neuroimaging studies have consistently shown that simulated touch with no inherent tactile content, such as touch observation^2–4^, working memory^5,6^, and imagery^4,7^, evoked neural activation in the somatosensory cortices. Previous functional magnetic resonance imaging (fMRI) studies have demonstrated that observed touch activates the primary (S1^3,8^) and the secondary somatosensory cortex (S2^2,9^). Although recent studies have provided evidence against this statement^10,11^, the involvement of the human somatosensory cortex for observed touch appears to be a plausible characteristic.

The involvement of somatosensory areas in tactile roughness perception is well documented by numerous physiological^12^ and neuroimaging studies^13,14^. For example, Kitada and colleagues observed various levels of activation in the parietal operculum when participants felt various rough surfaces. This finding suggests that this area is closely related to the perception of roughness magnitude^14^. On the other hand, only a few neuroimaging studies have so far investigated visually evoked neural representations of tactile intensities^15^. In the previous study by Morrison and colleagues, participants watched video clips showing a left arm being stroked by a brush at two different velocities inside the fMRI scanner^15^. They observed increased neural responses in posterior insular cortex when contrasting both stroke speeds. Their results demonstrate distinct neural representations of different tactile intensities even though the intensity information was perceived visually in absence of any tactile sensation. Another fMRI study at 7 Tesla monitored S1 activity while participants were watching video clips depicting their hand being passively touched by sandpapers of various intensities^16^. The results reveal the involvement of the posterior region of S1 in tactile discrimination by sight. Although these studies have shown intensity-dependent neural activations in the somatosensory cortical areas, they have compared neural activities elicited by only two tactile intensities, not multiple levels of intensity. Using two intensities may reveal neural activity for discriminating different tactile intensities, but not for encoding the intensity. Moreover, none of these studies explicitly investigated neural representations of visually evoked tactile intensities: The former primarily focused on neural responses associated with pleasant caressing speeds^15^ and the latter primarily compared neural responses during observed touch of self and others^16^. According to these previous observations, it is plausible that somatosensory cortices can represent multiple levels of perceived tactile intensities elicited visually. However, no study has directly compared brain activities in response to tactile and visually observed tactile stimulations with multiple intensities. Such a comparison can reveal how tactile information delivered via intra-modal and cross-modal channels is represented in the brain and whether there is a brain region encoding tactile information in a modality-independent manner.

Why then have neural representations of observed tactile intensities not been explored in more detail with more intensity levels? One possible reason may be the difficulty of creating visual stimuli that can effectively trigger the perception of rough textures with various intensities. In the aforementioned fMRI study^16^, approximately 20% of participants could not properly distinguish between roughness intensities by sight, even though the particle sizes of sandpapers were large enough for visual discrimination. To overcome the limits of conventional stimulations, we used an associative learning method in our study. Our participants learned to associate real and observed touch of sandpapers of different roughness levels. With associative learning, we expected that participants who had first experienced tactile roughness would recognize visually presented sandpaper of various roughness intensities more easily. This associative learning method has been successfully employed in fMRI studies in other domains^7,17^. Note that we used an association method to investigate whether active and observed touches of rough textures share the same neural representation. Therefore, the activity evoked by observed touch was induced by association learning was neither a vicarious nor an automatic neural response.

This study aimed to investigate the neural representations evoked by tactile explorations and visual observations of surfaces with various levels of roughness. The sandpapers used as stimuli had different roughness intensities, which were indicated by the color of their surfaces. During learning participants formed a strong association between a stimulus roughness intensity perceived by touch and its visual appearance (i.e. color). Using a multivoxel pattern analysis (MVPA), we first tested the feasibility of decoding visually evoked roughness intensity from brain activation patterns and identified brain regions containing multivoxel patterns with significant decoding performance. Then, these identified regions were compared with those obtained from the tactile explorations to uncover overlapping regions across sensory modalities. Furthermore, we also sought for a shared pattern of brain activity using a cross-modal classification method^18^. In the cross-modal classification, we trained a classifier on data from one modality and tested the data from the other modality. In particular, information-based searchlight MVPAs were used to look for brain regions exhibiting patterns of neural activity encoding perceived roughness intensities^19^. Once we identified brain regions, we examined each region to find whether misclassification patterns (i.e. pattern of confusion) from neural decoding were similar to the pattern of perceptual confusion from behavioral responses. Since MVPAs only assesses correct classification performance, an investigation of the confusion patterns could provide a richer description of the neural data^20^. In addition, comparing confusion patterns between neural and behavioral responses can help to find neural correlates of perceived roughness intensities.

## Results

### Summary

In this study, we investigated neural representations of perceived roughness intensities evoked by tactile explorations and visual observations. Using an associative learning method, we designed a methodologically novel fMRI paradigm that enabled us to test neural responses to five levels of tactile roughness intensity (0.3, 12, 40, 60, and 100 μm) delivered visually or by touch. We performed three searchlight MVPAs (a) on the data evoked by tactile explorations, (b) on the data evoked by visual observations, and (c) on the data evoked by both types of stimuli using the cross-modal classification method. In the case of (a), we decoded roughness intensity successfully from the multivoxel patterns in the anterior cingulate cortex (ACC) and the supramarginal gyrus (SMG). Case (b) showed that multi-level roughness information could be decoded from the bilateral posterior parietal cortex (PPC), the inferior occipital gyrus (IOG), and the primary motor cortex (M1) activity patterns, which were evoked by video clips that had been associated with tactile roughness intensities. This result demonstrates the feasibility of tactile intensity decoding from brain signals elicited by the mere observation of touch actions. In the case of (c), we found shared neural representations between tactile explorations and visual observations in the bilateral insular cortices, the SMG, and the ACC. Interestingly, the insular cortices were identified solely from the cross-modal classification analysis, suggesting a putative role of insular cortex in modality-independent tactile intensity encoding. In the follow-up correlation analyses, we derived a perceptual similarity matrix from the behavioral experiment and correlated it with neural similarity matrices (confusion matrices) of each identified brain region. Significant correlations were found only in the SMG.

### Behavioral data analysis

**Figure 1:**
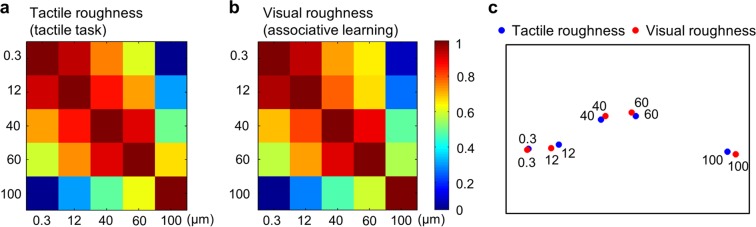
Results of the tactile task and associative learning sessions. Similarity matrices were computed based on the estimated difference of perceived roughness intensities between each pair of sandpapers (**a**) without and (**b**) with visual cues. These matrices were significantly correlated (*r* = 0.975, *p* < 0.001), suggesting that perceived roughness intensities were similar regardless of visual information. (**c**) The relative positions of five roughness intensities depicted in a 2-dimensional MDS map.

Figure 1 shows the perceptual similarity matrices of the uni-modal tactile task and the learning session. We measured a correlation between these two similarity matrices to statistically estimate how well perceived roughness intensities with and without visual information matched each other. The result of a Mantel test demonstrates that participants perceived tactile roughness intensities similarly regardless of the visual cues (*r* = 0.975, *p* < 0.01). The spatial organization of the five roughness intensities indicates that the sandpaper with particle size of 100 μm was recognized more easily than the other four intensities.

### Confirmatory analysis

In the fMRI visual runs, it is likely that participants perceived roughness intensities mainly from the learned association with the color of the paper strips instead of the visual appearance of the texture. Therefore, there is a possibility that brain signals could be evoked not only by tactile roughness information but also by low-level visual information. If that was the case, our MVPA results could be confined to the decoding of the low-level visual stimulation rather than the visually evoked roughness intensity decoding. To eliminate this potential ambiguity, we tested the following hypothesis: The brain signals are unlikely to contain low-level visual information if brain activation levels are correlated with the tactile roughness intensities, but not with color information.

We contrasted each of the visual observations of five roughness intensities against resting periods to seek brain regions activated in common by all visual stimuli. Random-effect group analyses (N = 15) revealed increased activations in somatosensory cortices comprising the inferior parietal cortices, S2, and the supplementary motor area. These contrasts also revealed the activations within early visual areas including the primary visual cortex along calcarine sulcus and lingual gyrus (Supplementary Tables S1–S5). We looked for the voxels identified in common among the five activation maps and found that in total 291 voxels of the four brain regions were overlapping: (1) 35 voxels in the right lingual gyrus, (2) 13 voxels in the right calcarine sulcus, (3) 38 voxels of the rolandic operculum extending to the S2 in the left hemisphere, and (4) 205 voxels in the left SMG. Of these activated brain regions, the lingual gyrus and the calcarine sulcus are part of the visual information processing network and S2 and SMG are part of the tactile information processing network^21^.

**Figure 2:**
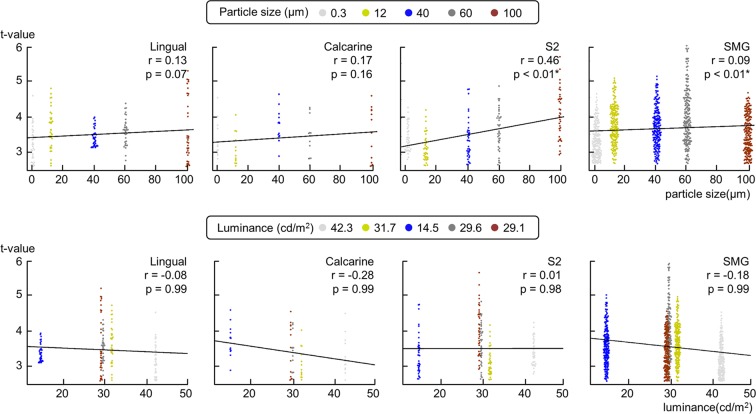
Results of the confirmatory analysis. To confirm that brain signals in the fMRI visual runs are mainly related to the associated tactile information and not to low-level visual stimulation, we conducted a confirmatory analysis. Using univariate GLM analyses, we found brain regions consistently activated across five roughness intensity conditions. Two visual (lingual gyrus and calcarine sulcus) and two somatosensory (S2 and SMG) regions were identified. Next, we correlated the activation levels (t-value) with the tactile roughness information (particle size) and the low-level visual information (luminance) for each identified cluster. Significant correlations were found only in the somatosensory regions when correlating t-values with particle sizes. Therefore, these results confirmed that neural signals in the visual runs contain relevant associated tactile information, not low-level visual information.

We investigated how regional activation levels (t-values) for each identified cluster varied with low-level visual stimulation (luminance) and tactile roughness information (particle size), respectively. Between t-values and particle sizes, significant correlations were found in the S2 (*r* = 0.46, *p* < 0.01) and SMG (*r* = 0.09, *p* < 0.01), but not in the visual areas (Fig. 2). In contrast, between t-values and luminance, no significant correlation was found in any of the identified clusters (all *r*s < 0.28, *p*s > 0.98). Overall, we observed significant correlations solely in brain regions processing tactile information, even though the brain signals were elicited by the visual stimuli. Therefore, this result confirmed the hypothesis that neural activities elicited by the visual observation of tactile roughness intensities were mainly related to the associated tactile information, not to the low-level visual information.

### Functional imaging data analysis

The purpose of searchlight MVPA was not only to identify brain regions allowing discrimination of the five roughness intensities, but also to explore the correspondence between brain activity patterns when roughness intensities were presented in different modalities. To this end, we performed three different searchlight analyses.

**Figure 3:**
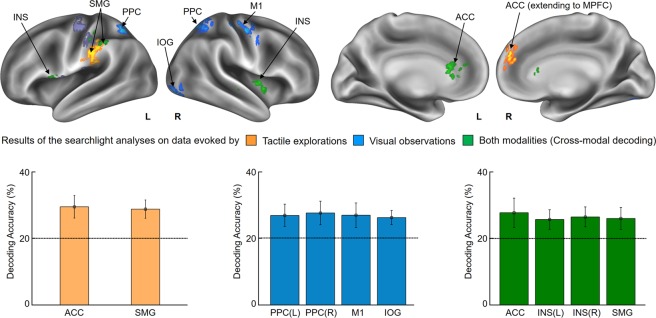
Results of the whole-brain searchlight analyses. Three searchlight analyses identified voxel clusters exhibiting patterns of neural activity carrying information for roughness intensity. The clusters identified from tactile exploration, visual observation, and both modalities are colored in orange, blue, and green, respectively. In addition, activated brain regions evoked by tactile explorations using a univariate GLM contrasting tactile stimulations (including all five roughness intensities) against resting periods are highlighted in purple. Significant activations were found in the contralateral M1 and S1 regions (p < 0.001 uncorrected, size > 50). The lower part of the figure shows the classification performances of each identified cluster. The graphs are shown in the colors specified above. Chance level is indicated by the dashed line (20%) and error bars indicate standard errors. Abbreviations: ACC, anterior cingulate cortex; SMG, supramarginal gyrus; PPC, posterior parietal cortex; M1, primary motor cortex; S1, primary somatosensory cortex; MPFC, medial prefrontal cortex; IOG, inferior occipital gyrus; INS, insular cortex.

The first searchlight analysis, which was performed on data evoked by tactile explorations with the right finger, found two significant clusters (*p* < 0.001 uncorrected, size > 50) located in the ACC extending to the medial prefrontal cortex (MPFC) in the ipsilateral hemisphere and the SMG in the contralateral hemisphere (Fig. 3 and Table 1). Decoding accuracies obtained from each identified cluster were as follows (presented as mean ± standard deviation for each cluster): 29.4 ± 3.4% for the ACC; and 28.7 ± 2.7% for the SMG. The second searchlight analysis, which was performed on data evoked by visual observations, identified four significant clusters located in the bilateral PPC, the IOG including primary visual cortex (V1), and the M1 in the ipsilateral hemisphere (*p* < 0.001 uncorrected, size > 50). Decoding accuracies obtained from each identified cluster were as follows: 26.7 ± 3.3% for the contralateral PPC; 27.4 ± 3.5% for the ipsilateral PPC; 26.8 ± 3.6% for the M1; and 26.0 ± 2.1% for the IOG. The third searchlight analysis employed a cross-modal classification method to uncover neural representations of perceived roughness independent to the sensory modalities. The classifier was trained on data elicited by tactile explorations and tested on data elicited by visual observations, and vice versa (see Materials and Methods). As results, the bilateral insular cortices, the SMG in the contralateral hemisphere, and the ACC in the ipsilateral hemisphere were identified (*p* < 0.001 uncorrected, size > 50). Decoding accuracies obtained from each identified cluster were as follows: 27.6 ± 4.4% for the ACC; 25.6 ± 2.9% for the contralateral insula; 26.4 ± 2.9% for the ipsilateral insula; and 26.0 ± 3.3% for the SMG. These values were averaged across the two folds of cross-modal classification, i.e. an average of tactile-to-visual and visual-to-tactile cross-classifications. Thus, we further investigated whether these regions discriminated roughness intensities with a similar level of performance for each fold separately. As a result, all identified brain regions showed high classification performance for both folds (Supplementary Table S6). The bootstrap procedure demonstrated that the obtained clusters were unlikely to have been found by chance^22^. The probability of obtaining a cluster as large as ours was less than 0.05.

**Table 1:** Identified brain regions in which the local activity patterns allowed to discriminate the roughness intensities (*p* < 0.001 uncorrected, size > 50).

Regions	Side	MNI coordinates			Cluster size	*T*	*Z*
		x	y	z			
**Tactile exploration**							
Anterior cingulate cortex	Right	2	52	12	192	8.99	5.10
*Medial prefrontal cortex*	Right	0	44	26		6.87	4.47
—	Right	12	50	24		6.59	4.38
Supramarginal gyrus	Left	−50	−26	16	382	8.20	4.89
—	Left	−58	−38	12		7.51	4.68
—	Left	−60	−24	40		7.42	4.65
**Visual observation**							
Posterior parietal cortex	Left	−24	−52	50	201	6.69	4.41
—	Left	−30	−52	60		5.88	4.11
—	Left	−20	−60	44		5.08	3.77
Precentral gyrus	Right	40	−12	48	171	6.57	4.37
—	Right	46	−6	42		6.38	4.30
*Postcentral gyrus*	Right	44	−24	56		5.92	4.12
Posterior parietal cortex	Right	34	−48	58	216	5.69	4.03
—	Right	22	−56	56		5.68	4.03
—	Right	16	−62	56		5.18	3.82
Inferior occipital gyrus	Right	34	−86	−6	134	5.41	3.91
—	Right	34	−74	−6		5.34	3.88
**Cross**-**modal decoding**							
Anterior cingulate cortex	Left	−14	32	10	193	7.27	4.61
—	Left	−10	32	2		7.05	4.53
—	Left	−8	28	14		6.39	4.30
Insula	Right	50	2	2	98	7.07	4.54
—	Right	40	−2	−2		6.18	4.22
Supramarginal gyrus	Left	−52	−30	40	74	6.20	4.23
*Inferior parietal cortex*	Left	−56	−38	48		5.93	4.13
—	Left	−48	−36	52		5.42	3.92
Insula	Left	−44	12	−2	61	6.04	4.17
—	Left	−46	2	−4		5.89	4.11

Cluster size indicates N voxels, T indicates peak t-values, Z indicates peak z-values. Entries without brain region labels or in italics indicate sub-peaks within the cluster named above.

### Correlation analysis

**Figure 4:**
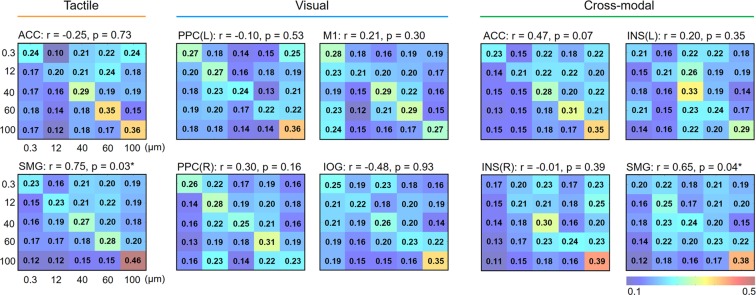
Confusion matrices for fMRI decoding in identified clusters. The rows of the matrix indicate the actual roughness intensity provided to the participants and the columns indicate the predictions by a neural classifier. An ideal confusion matrix would have ‘1’ on the diagonal (correct classifications) and ‘0’ in the off-diagonal entries (errors). Correlations of off-diagonal entries between behavioral similarity matrix and neural confusion matrix were calculated. Resulting correlation coefficients and significances are shown above each matrix. Significant correlations (indicated by *) were found in two SMG clusters (*p* < 0.05).

Having found brain regions carrying information for roughness intensity discrimination as shown above, we further explored the relationship between behavioral and neural representations of perceived intensities. We achieved this by comparing the pattern of confusions^20^. Since confusions in the tactile task occurred mainly for similar levels of roughness intensity (i.e. 0.3 and 12 μm levels, Fig. 1), we expected the same tendency from the fMRI decoding results as well. First, we extracted confusion matrices for each searchlight cluster (Fig. 4). In Fig. 4, the values on a row *j* and column *k* in each matrix represents a probability that the presentation of roughness *j* was predicted as roughness *k*. The diagonal entries are for correct classifications and all off-diagonal entries are for misclassifications: thus, an ideal confusion matrix would contain ‘1’ everywhere on the diagonal and ‘0’ in the off-diagonal entries. The confusion matrices obtained from each cluster were then correlated with a behavioral similarity matrix. In particular, we computed correlation coefficients between off-diagonal elements of behavioral and neural confusion matrices. Among the 10 identified clusters, we found two significant correlations, both of which were found in the contralateral SMG (all *p*s < 0.05; *r* = 0.75 for the searchlight cluster identified from the tactile explorations and *r* = 0.65 for the searchlight cluster from the cross-modal classification) (Fig. 4). Thus, confusion matrices in SMG showed patterns similar to the behavioral responses regardless of the stimulus modalities. All other searchlight clusters did not show any significant correlation (all *p*s > 0.07, *r*s < 0.48). It is also noticeable that the highest decoding accuracies in each confusion matrix were mostly found when the sandpaper with particle size of 100 μm was presented (observed in 7 out of 10 matrices). This observation is in line with the behavioral responses (Fig. 1).

To examine whether these group-level results (N = 15) were consistent with individual-level results, we carried out the same correlation analysis in each identified cluster using single subject data (Supplementary Tables S7–S9). In the ACC and SMG, which were identified from the searchlight analysis of tactile explorations, zero and seven (all *p*s < 0.05; *rs* > 0.46) participants showed significant correlations, respectively. In the left PPC, right PPC, IOG, and M1, which were identified from the searchlight analysis of visual observations, one (*p* = 0.05; *r* = 0.55), zero, one (*p* = 0.05; *r* = 0.59), and one (*p* = 0.04; *r* = 0.59) participant showed significant correlations, respectively. In the ACC, SMG, left insula, and right insula, which were identified from the searchlight analysis of cross-modal classification, two (both *p*s < 0.05; *rs* > 0.45), six (all *p*s < 0.05; *rs* > 0.47), one (*p* = 0.03; *r* = 0.63), and two (both *p*s < 0.03; *rs* > 0.55) participants showed significant correlations, respectively.

## Discussion

The present study aimed to explore how the human brain encodes roughness intensities perceived by tactile explorations and learning-based associated visual observations. Using searchlight MVPAs, we tested whether roughness intensities could be decoded from brain signals elicited from (a) tactile explorations, (b) observation of tactile explorations, and (c) both sensory modalities. Case (a) revealed successful decoding of roughness intensity in the ACC and the SMG and case (b) identified the bilateral PPC, the IOG, and the M1. Case (c) found significant decoding performances in the bilateral insula, the SMG, and the ACC. In the follow-up correlation analyses, we correlated the perceptual similarity matrix with the neural similarity matrices of each identified region. Interestingly, significant correlations were found only in the SMG.

### Tactile and visual responses to surface roughness intensity

Our searchlight analysis revealed that the SMG and the ACC were implicated in the discrimination of tactile roughness intensities. However, we did not observe any activation in S1, which is known as a main sensory receptive area for the sense of touch^23^. Our observation conforms to the hierarchical view of the human somatosensory system^24^. According to this view, tactile information is relayed from the thalamus to S1 and then distributed to adjacent cortical regions including the SMG for higher level processing^24,25^. We thus speculate that these results reflect the different roles of the SMG (high-level tactile processing, such as stimulus discrimination) from the S1 (low-level tactile processing, such as stimulus detection). Consistent with this perspective, a human fMRI study using vibrotactile stimulations showed that discrimination of stimulated location evoked activations in SMG but not in the S1, while detection of tactile stimulus evoked S1 activity but no SMG activity^26^. We also found significant decoding performance in ACC, but this might be related to the processing of affective pain^27^. We will discuss in detail in the next section.

Although a number of studies have reported somatosensory cortex activations during touch observation, it is still debated which brain region is mainly associated with observed touch processing, e.g. S1^3,8,28^, S2^2,9^, and PPC^11^. On one hand, Schaefer and colleagues found a significant increase of activity in S1 by contrasting brain activities evoked by seeing a hand being touched against those evoked by seeing a hand not being touched^28^. On the other hand, Keysers and colleagues observed neural activity changes in S2 when participants watched tactile stimulations on legs^2^. A more recent study with a large number of participants showed that touch observations primarily activated the PPC, *not* the S1 or S2^11^. In line with the last study, our searchlight analysis demonstrated that the bilateral PPC encoded visually evoked tactile information. Although we observed activations in ipsilateral S1 (Table 1), we cannot confirm this S1 engagement, given that the majority of previous studies have reported brain activations in contralateral S1^11,16^. Therefore, among the three brain regions mentioned above, our results supported a hypothesis that the PPC plays a role in information processing of observed touch.

Significant decoding performance in IOG is also an interesting observation. It is known that visual texture and shape information are processed separately in the ventral stream^29,30^. For instance, Cant and Goodale visually presented objects with various textures and shapes to participants during fMRI signal acquisition^30^. Their results showed that visual texture information was processed in the IOG and collateral sulcus (CoS), while visual shape information was processed in the lateral occipital complex (LOC). Similar to this finding, our results showed an involvement of the IOG in visual texture information processing.

### Shared neural representations in insular cortices

One of the key findings in this study is that the cross-modal classification analysis revealed successful decoding in insular cortices. Human neuroimaging studies have suggested the insula as an essential brain region for multimodal sensory processing (e.g. visual-tactile^31,32^; auditory-visual^33^). For instance, Gentile and colleagues investigated brain activation during visual-tactile integration tasks and revealed that the insula was one of the main brain regions binding information from the multiple sensory modalities^32^. Moreover, positron emission tomography (PET) studies have demonstrated the involvement of insular cortices in cross-modal processing of visuotactile information^34,35^. In agreement with these previous functional imaging studies, our results provide additional evidence that the insula is crucial for the integration of visual and tactile information.

Our results successfully showed that insular cortices encoded roughness intensities shown in the video clips in patterns similar to the actually felt intensities, which can be considered as neural evidence for a shared neural representation with respect to touch sensation. The modality-independent property of insular cortices has been supported by several human somatosensory studies^3,15^. For example, Morrison and colleagues provided convincing evidence of a shared neural representation in insula between felt and observed touch^15^. Their participants saw video clips showing the arm of another person being stroked. The authors compared their visually evoked activation patterns with the activations from felt touch and similar uni- and multi-variate responses were observed in the insula. Based on this observation, they argued that the posterior insular cortices carry a shared representation of touch sensation. Together with these findings, our results support the hypothesis that the modality-independent neural representation for touch sensation is located in the insular cortices. It is noteworthy that insular cortices were not identified by single-modal decoding analyses. Although further investigations are needed, this suggests that uni- and supra-modally encoded roughness information are characterized by different neural activity patterns.

In addition to the bilateral insular cortices, our cross-modal classification analysis found significant decoding performance in ACC and SMG. Interestingly, all of the identified brain regions are well known as substantial parts of the brain networks processing pain generated by nociception^36,37^. Accordingly, our observation leads us to form another hypothesis, namely that the neural encodings in those regions are attributed to affective pain processing. A number of neuroimaging studies have suggested that somatic and vicarious pain activations include overlapping brain areas^38,39^. Corradi-Dell’Acqua and colleagues examined the brain responses evoked by somatic and vicarious pain and found shared neural representations in the bilateral insula and ACC^38^. Moreover, they claimed that the identified common neural patterns in these areas were not specific to pain, but also reflected a more general aspect of negative emotional experience. In the current study, participants explored fine-textured and harmless sandpapers during the functional image acquisition. However, we cannot rule out the possibility that the rough stimuli could evoke a complex feeling, rather than a simple tactile sensation. Perceived roughness might have aroused a negative emotion or some discomfort to the participants, which might be reflected in the activations of insula, ACC, and SMG.

### Neural activity patterns associated with roughness intensity perception

We further investigated how neural activity patterns are related to behavioral tactile roughness perception using correlation analyses of confusion matrices (i.e. off-diagonal elements of neural and behavioral similarity matrices). Methodologically similar to our study, previous MVPA studies showed that the examination of confusion patterns could provide a richer description than mere decoding performance^20,40^. In the current study, we found that neural activity in the SMG was closely associated with the behavioral data. Remarkably, significant correlations were found between the SMG activation and behavior independent of the sensory cues. Numerous studies have suggested that the neural activities in the SMG were related to the behavioral performance in tactile tasks^41–43^. A previous study observed that the SMG was strongly activated during temporal order judgment tasks of tactile stimulations and suggested an important role of the SMG in behavioral tactile discriminations^42^. In addition, a case study of a right parietal damaged patient demonstrated a close functional connection of the SMG to somatosensory perception^43^. Combining evidence from the present and the previous findings, we suggest that neural activity in the SMG is more closely related to human tactile perceptual discrimination than the activities in other brain regions.

### Limitations

In the fMRI visual runs, participants perceived roughness intensities from the association with the color of sandpapers instead of texture of the paper strips itself. In other words, they did not perceive roughness intensity (i.e. particle size) directly. Nonetheless, our confirmatory analysis demonstrated that neural data contained relevant tactile information, not low-level visual information. We therefore argue that associative learning could be an effective way to evoke tactile sensation via visual stimuli when tactile sensations are not easily recognizable by sight. However, our argument could have been more solid if we had also examined the neural activity patterns evoked by other colors not associated to any roughness levels. For example, if we had visually presented participants sandpapers with colors other than the five associated colors and compared the result with the present one, we could have explicitly assessed the effects of the associative learning methods. In a similar vein, a previous tactile imagery study successfully eliminated the influence of visual cue presentation by virtue of presenting a meaningless visual cue as a control condition^7^. This will be investigated in our next study.

The decoding accuracy level seems low compared to other tactile decoding studies. For example, Beauchamp and colleagues obtained 75% of decoding accuracy for the 2-level discrimination task (stimulus on left vs. right hand, chance performance was 50%)^44^. However, it should be acknowledged that the tactile intensity decoding as performed in our study might be more challenging than the tactile stimulus site decoding, because the brain areas specializing in different parts of the body are well-developed (e.g. cortical homunculus), while it still remains an open question how the human brain encodes tactile intensity levels. In the present study, our statistical tests successfully verified that the decoding accuracies for each region were significantly above chance, even though performances do not seem very high.

Using the data from only 6 fMRI runs might not be enough to validate the decoding performance. To alleviate this problem, in our classification analysis, we modeled individual regressors for each trial of each fMRI run in order to increase the number of input features for a searchlight analysis. The generated 150 regressors (25 regressors per run) were then used to build and test a classifier for each participant. It may still not provide enough data to validate decoding outcomes completely, but previous cross-modal studies have also reported decoding results with less than 3 fMRI runs per modality^18,45,46^. Touch experiments require very long sessions, our total experiment took approximately two hours, thus increasing the number of fMRI runs was not feasible.

## Materials and Methods

### Participants and ethics approval

Fifteen healthy volunteers (9 females, average 26.7 ± 3.6 years old) with no contraindications against MR investigations and no history of neurological disorders participated in the experiment. All participants were right-handed and had no deficits in tactile processing. Experimental procedures were approved by the ethical committee of the University Clinics Tübingen (649/2016BO2) and the study was conducted in accordance with the Declaration of Helsinki. All participants were informed about the experimental procedure and gave informed consent prior to their participation.

### Stimuli

#### Tactile stimuli

**Figure 5:**
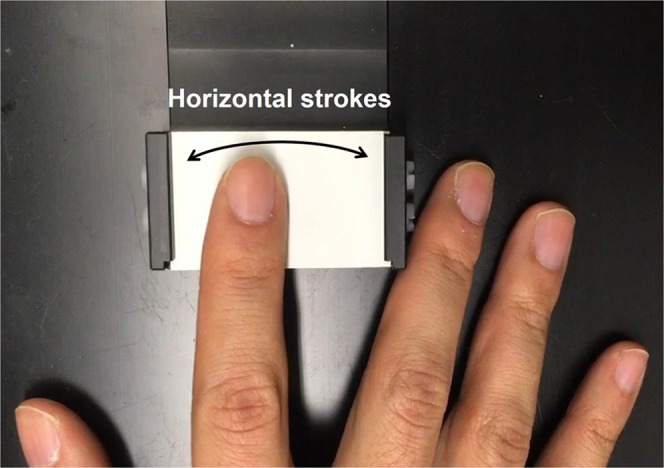
Tactile exploration of a sand paper (the white color indicates a texture with particle size of 0.3 μm). This is a screenshot from one of the videos used for visual roughness stimulation. The arrow indicates the finger motion used to explore the surface.

Sandpapers (aluminum-oxide abrasive paper, 3 M Center, St. Paul, MN, USA) with ten different roughness levels were prepared in the size of 5 × 3 cm^2^ and attached on a black plastic plate sized 5 × 8 cm^2^. These sandpapers were successfully used and validated in a previous fine surface texture perception study^47^. The average particle sizes assigned by the manufacturer were 0.3, 1, 3, 5, 9, 12, 30, 40, 60, and 100 μm where a larger particle size formed a rougher surface and each particle size were indicated by a different surface color (Fig. 5).

A pilot experiment was conducted to select stimuli delivering distinct roughness intensities. Five volunteers (2 females, average 27.8 ± 3.0 years old) participated in this pilot experiment and were presented each sandpaper strip five times. After explorations with their right index fingertip, participants were asked to rate the perceived roughness intensity on a scale of 0 (very smooth) to 100 (very rough). A one-way ANOVA revealed that roughness intensity was perceived differently across the particle sizes (F_9,240_ = 145.49, *p* < 0.01) and post-hoc t-tests revealed four distinct roughness intensity groups (all *p*s < 0.01): (1) 0.3, 1, 3, 5, 9, 12, (2) 30, 40, (3) 60, and (4) 100 μm. One particle size was chosen in each group as stimulus: 12, 40, 60, and 100 μm. Moreover, we added the 0.3 μm stimulus so as to investigate the influence of under threshold roughness, based on the previous report that the absolute detection threshold for roughness was approximately 1 μm in particle size^47^. Thus, five roughness intensities (0.3, 12, 40, 60, and 100 μm, in the colors white, yellow, blue, gray, and brown) were selected for the main experiment.

#### Visual stimuli

Video clips of tactile explorations were recorded with the resolution of 1920 × 1080 at 60 frames per second. Each clip displayed a right hand exploring one of the five sandpaper strip by stroking it horizontally with the index finger at a constant speed. To reduce a potential gender effect, video clips were recorded for each roughness level once with a male and once with a female hand. In the experiment, video clips showing tactile explorations by female’s hand were presented to female participants and vice versa. To create an association between participants’ perceived tactile roughness intensities and participants’ view on their own sandpaper explorations, the visual information provided in the clip was as similar as possible to their visual input when participants were actually exploring the surface texture. The recording video camera was positioned at a distance of 20 cm from the stimulus surface toward the participant and 30 cm above from the tabletop, which was close to the point of view of a participant. All videos were recorded in a fixed position and a constant illumination level was provided. Each video clip lasted 3 s and did not contain any auditory contents.

### Experimental design

**Figure 6:**
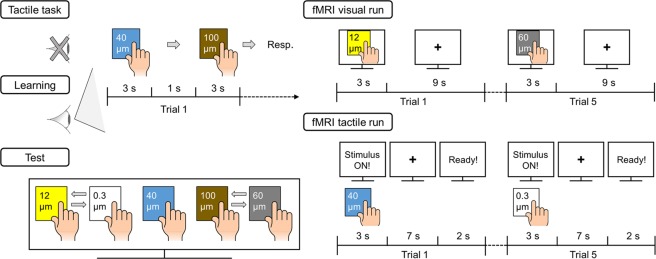
Schematic overview of the experimental design. Participants took part in tactile task, associative learning, and association test sessions outside of MR room. In the tactile task session, participants responded the perceptual dissimilarity between two roughness intensities after exploration of sandpaper pairs without visual cues. In the associative learning session, participants performed the same tasks, but they observed their surface explorations with their eyes. The associated level between perceived roughness intensities and their view on sandpaper explorations was tested before fMRI scanning sessions. Functional images were acquired in six runs, with three runs for visual and tactile explorations, respectively. Participants viewed video clips showing finger movements on different colors of sandpaper in the fMRI visual runs, while participants actually explored the sandpapers following to the visual instruction on the screen in the fMRI tactile runs.

To minimize confounding factors due to finger movement variations across participants, participants performed a training session to standardize their finger movements. The main experiment consisted of four sessions: (1) Tactile task, (2) associative learning, (3) association test, and (4) fMRI scanning (Fig. 6). Tactile task, associative learning, and association test sessions were conducted outside of the MR room.

#### Tactile task

Participants used an eye mask to block visual information and completed five blocks of 20 trials (counterbalanced pairwise combinations of five stimuli: _5_P_2_ = 20 trials). In each trial, two sandpapers were presented one after the other and participants explored each with their fingertip. Maximum exploration duration was 3 s per stimulus and there was a 1 s inter-stimulus interval. Thereafter, participants were asked to rate perceived dissimilarity between two sand papers and verbally reported their rating in a free modulus scale. This method was chosen to reduce any bias due to different internal scales across participants^48^. Pair presentation was randomized in each block. The responses obtained from the first block were not used in the data analysis (considered as a practice block).

#### Associative learning

The procedure of the associative learning session was identical to that of the tactile task session, except that participants were not wearing a mask. Therefore, they could form an association between the perceived roughness of the sandpapers and their appearance. More specifically, they could associate the tactile roughness of a sandpaper strip with its color, surface properties and finger movement difference triggered by the roughness intensity levels. However, participants were not likely to visually recognize roughness level based either on finger movement difference or on particle size. Recall that all sandpapers were finely textured and it was difficult to distinguish particle sizes with the naked eyes. Therefore, the color-coding of roughness intensity played a central role in the associative learning between tactile and observed touch.

#### Association test

First, participants viewed the five video clips on a wide 27-inch monitor (Dell, 60 Hz). Next, they were asked to arrange on the screen all clips in an increasing order of perceived roughness intensity. The correct order was shown at the end of the test. If participants failed to arrange the clips in the correct order, they repeated one learning block and were tested again until responding correctly. This procedure guaranteed that every participant had a sufficient level of association before taking part in the fMRI scanning session. Only one participant needed one more learning block.

#### fMRI scanning

Participants laid in a supine position with their right arm comfortably placed along the magnet bore. They wore earplugs and watched a computer screen with a projector resolution of 1280 × 1024 pixels at a refresh rate of 60 Hz via an angled surface-mirror. Functional MRI data were acquired in six runs, with three runs for visual observations and three for tactile explorations, respectively. Visual and tactile blocks were tested one after another. 8 participants started with the visual block and 7 participants started with the tactile block. Each run started with a 10 s baseline period. Then, 25 trials were presented in five blocks of five trials (5 repetitions × 5 roughness intensities). Roughness intensities in each block were presented in a randomized order. In the visual observation runs, each trial consisted of a stimulation period of 3 s followed by a fixation resting period of 9 s. In the stimulation period, participants were asked to recall the associated perceived roughness intensity while they watched a video clip. In the tactile exploration runs, participants rested their right index finger on the MR table for 7 s before a stimulus was given. When ‘Ready!’ was displayed at the center of the screen, they placed their finger on the given sandpaper and maintained the pose for 2 s. Then, when ‘Stimulus ON!’ was displayed, participants explored the sandpaper surface for 3 s with the same motions as they performed in the associative learning session (moving their right index fingertip from side to side). After each tactile exploration, participants slightly lifted their finger while an experimenter exchanged the stimulus. For both types of run, the duration of each trial was 12 s and each run lasted 5 min 10 s.

### MR data acquisition and preprocessing

Neuroimaging data were acquired on a 3 Tesla Siemens Prisma system with a 64-channel head coil (Siemens Medical Systems, Erlangen, Germany). Anatomical images were obtained using a T_1_-weighted sequence (ADNI, 192 slices) with the following parameters: repetition time (TR) = 2,000 ms, echo time (TE) = 3.06 ms, flip angle = 9°, field of view (FOV) = 256 mm, and voxel size = 1 mm³. Functional images were acquired using a slice-accelerated multiband gradient-echo-based echo planar imaging (EPI) sequence using T_2_^*^-weighted blood oxygenation level dependent (BOLD) contrast (multiband acceleration factor: 2): 46 slices, TR = 1,520 ms, TE = 30 ms, flip angle = 68°, FOV = 192 mm, slice thickness = 3 mm, and in-plane resolution = 3 × 3 mm^2^. The functional images covered the whole cerebrum. Standard preprocessing of the fMRI data was performed using SPM8 (Wellcome Department of Imaging Neuroscience, UCL, London, UK) and a high-pass filter of 128 s was used to remove low frequency noise. The EPI data were corrected for slice-timing differences, realigned for motion correction, co-registered to the individual T_1_-weighted images, normalized to the Montreal Neurological Institute (MNI) space, and spatially smoothed by a 2-mm full-width-half-maximum (FWHM) Gaussian kernel.

### Data analysis

#### Behavioral data analysis

Since the behavioral responses of perceptual differences between two roughness intensities were recorded in different subjective scales, these were normalized by a two-step procedure^49^. First, a geometric mean (M_p_) of subjective responses over all conditions for each participant and a grand geometric mean (M_g_) over all conditions and participants were calculated. The response of each participant was multiplied by the scaling factors, M_n_ = M_g_/M_p_. Next, to scale the values between 0 and 1, the minimum value of the responses was subtracted from each of the responses and the resulting value was divided by the new maximum for each participant. These normalized behavioral responses were averaged across all participants to obtain a group-level dissimilarity matrix. An entry at the *j*-th row and the *k*-th column of the dissimilarity matrix was the average perceived difference in a normalized scale when the *j*-th intensity was presented followed by the *k*-th intensity (*j*, *k* = 1, …, 5). We then applied a multidimensional scaling (MDS) to this dissimilarity matrix to yield a spatial organization of perceptual responses for the five different roughness intensities. For MDS plots calculated for the tasks, the Kruskal’s stress values representing the goodness of fit were less than 0.01. Regarding a rule of thumb for a stress value^50^, Kruskal stated that “Our experience with experimental and synthetic data suggests the following verbal evaluation: 20% poor, 10% fair, 5% good, 2.5% excellent, 0% perfect.”. Since our stress value of 2-dimensional spaces was less than 0.01 (1%), we considered that 2-dimensional spaces were sufficient to describe the behavioral data.

#### Confirmatory analysis

We first measured the luminance of the color of sandpapers in each presented video clips with a UDT S370 photometer (Graseby Optronics, Orlando, FL). Displayed mean luminances of the surface colors were 42.3, 31.7, 14.5, 29.6, and 29.1 for the white (particle size: 0.3), yellow (particle size: 12), blue (particle size: 40), gray (particle size: 60), and brown (particle size: 100), respectively. Since luminance is a measure to describe the perceived brightness of a color^51^, we assumed that luminance could characterize this low-level visual information. Moreover, a previous fMRI study has demonstrated the effects of luminance of visual stimuli on BOLD responses in human primary visual areas^52^. In particular, a significant increase in the neural activities was observed as the luminance increased. The confirmatory analysis consisted of two steps. First, univariate general linear model (GLM) group analyses were carried out to search for brain areas showing significant BOLD signal changes between the visual observation and the resting conditions. Specifically, we performed five GLM analyses contrasting visual observation on each of roughness intensities against resting periods, e.g. 0.3 (particle size) – resting, 12 – resting, 40 – resting, etc. Then, the common brain regions activated by the five different intensities were obtained. Second, we examined how neural activation levels (i.e. t-values) within each of the identified brain regions correlated with luminance or particle size. The resulting correlation coefficients were used to test our hypothesis.

#### Functional imaging data analysis

Our multivariate analysis aimed to identify brain regions representing roughness intensity information according to the stimulus modality: Visual observations (viewing video clips associated with perceived tactile intensities) and tactile explorations (exploring different intensities of surface roughness). To this end, we performed volume-based searchlight MVPAs to seek local neural activity patterns that would allow successful classification of the five roughness intensities.

We first used a GLM to convert functional imaging data into data for classifier training and testing in searchlight MVPAs. Specifically, standard predictors were made by the convolution of a box-car function of the stimulus ‘on’ periods with a hemodynamic response function (HRF) implemented in SPM8. Each regressor modeled an individual trial in order to increase the number of examples. As results, a total of 150 regressors (6 fMRI runs × 5 blocks × 5 trials) were built to model the data for each participant. Obtained parameter estimates were used as input features for a searchlight analysis implemented in the Searchmight Toolbox^53^. We constructed a searchlight consisting of a center voxel and its neighborhood within a 3 × 3 × 3 voxels cube. In each searchlight, a 5-class Gaussian Naïve Bayes (GNB) classifier decoded the spatial pattern of the parameter estimates in the voxels into one of the five roughness intensity categories. We performed three searchlight analyses: (a) analysis on data evoked by tactile explorations, (b) analysis on data evoked by visual observations, and (c) analysis on data evoked by both stimuli types. In cases of (a) and (b), each experimental run was considered as one-fold and a 3-fold cross-validation procedure was performed. The resulting decoding accuracies were stored in the center voxel of the searchlight cube. Chance-level accuracy (0.2 in this case) was subtracted from the value stored in each voxel to yield deviations from chance. A random-effects group analysis was then performed on these individual accuracy maps to identify commonalities among individual neural activity patterns. This test was implemented as a one-sample *t* test against 0 to identify above-chance decoding accuracy in the MVPA. In case of (c), all analysis steps were identical except for the cross-validation procedure. Here, data elicited by tactile explorations were labeled as ‘Group 1’ and data elicited by visual observations were labeled as ‘Group 2’. In the first step, the classifier was trained on ‘Group 1’ data and tested on ‘Group 2’ data. In the second step, ‘Group 2’ data were used for training and ‘Group 1’ data for testing (a cross-modal classification method). The resulting decoding accuracies from both cross-validation steps were averaged and assigned to the center of the searchlight cube. This method ensured that the classifier could correctly decode the roughness intensities only if a shared pattern of neural activity was evoked by tactile explorations and visual observations.

To correct the searchlight results for multiple comparisons, we estimated an empirical cluster size threshold for a group of participants in searchlight accuracy maps. Following the sign-flipping permutation procedure^22^, a cluster size obtained from our group analysis was compared with a reference distribution of a cluster size that one would obtain by chance. Under the null hypothesis, searchlight accuracy values would be distributed between −0.2 and 0.8 after chance level subtraction. Namely, under the null hypothesis, the sign of the searchlight accuracies would be ‘+’ or ‘−’ with a probability of 80% and 20%, respectively. To estimate how large clusters could be formed under this condition, we randomly flipped the sign of the searchlight accuracy maps of a random number of participants. These maps were then considered as group samples under the no-effect condition. Then, a random-effect analysis on these maps was performed to assess the size of the biggest cluster. The null-distribution of cluster sizes was yielded by 1,000 repetitions of this procedure. In this study, we reported the clusters in the 5% of the upper tail as significant, i.e. *p* < 0.05 corrected for multiple comparisons via cluster size.

#### Correlation analysis

If certain stimuli are more similar to each other than others, humans would show higher confusion rates for these stimuli in their behavioral response as well as in fMRI decoding from neural activities that contribute to their behavioral decision^20^. Inspired by this notion, the identified clusters from the searchlight analyses were further investigated to find which brain areas showing the identified clusters were more closely related to the perception of roughness intensity in behavioral responses. We examined whether a correlation existed between confusion patterns of behavioral and neural similarities. As an indicator for behavioral similarity, we created a similarity matrix by subtracting each value of the behavioral dissimilarity matrix from 1. As an indicator for neural similarity, we averaged the confusion matrices of each participant for each identified cluster. Then, we employed the Mantel test to calculate a correlation between the behavioral and neural similarity matrices^40^. The significance of the test was determined from the probability distribution obtained by 10,000 times of repeated permutations. For each permutation, we permuted the values of each similarity matrix and determined the expected distribution of the statistics under the null hypothesis. The probability of the observed correlation arising by chance was then yielded by observing where the statistic calculated from the observed data fell in the permuted distribution. Together with the group-level analyses, we performed the identical correlation analyses at the single subject level.

## Supplementary information

Supplementary information

## Data Availability

Both the original and processed fMRI images related to this publication will be available to share upon request with a legitimate reason such as to validate the reported findings or to conduct a new analysis (Junsuk Kim; junsuk.kim@skku.edu).
