# Supplementary material for: Shared neural representations of tactile roughness intensities by somatosensation and touch observation using an associative learning method
Source: Sci Rep. 2019 Jan 11;9:77. doi: 10.1038/s41598-018-37378-w (PMC6329784; doi:10.1038/s41598-018-37378-w)
Supplement: Supplementary file 1 — Supplementary information [file 41598_2018_37378_MOESM1_ESM.pdf]

Supplementary Information

Shared neural representations of tactile roughness intensities by somatosensation and touch observation using an associative learning method

Junsuk Kim[1,2,3], Isabelle Bülthoff[1], Sung-Phil Kim[4,*], and Heinrich H. Bülthoff[1,*]

[1]Department of Human Perception, Cognition and Action, Max Planck Institute for Biological Cybernetics, Tübingen, 72076, Germany

[2]Center for Neuroscience Imaging Research, Institute for Basic Science (IBS), Suwon, 16419, Republic of Korea

[3]Department of Biomedical Engineering, Sungkyunkwan University, Suwon, 16419, Republic of Korea

[4]Department of Human Factors Engineering, Ulsan National Institute of Science and Technology, Ulsan, 44919, Republic of Korea

Supplementary Tables S1-S5. Activated clusters from contrasting analysis using univariate group GLM (p < 0.001 uncorrected, cluster size > 30). Entries without the brain regions name-labels indicate sub-peaks within the cluster named above them. *T* indicates peak t values; *Z* indicates peak z values.

Visual observation of roughness intensity ‘0.3’ – Resting

Regions	Side	MNI coordinates			Voxels	*T*	*Z*
		x	y	z			
**Lingual gyrus**	**Right**	**18**	**-84**	**-2**	**42**	**5.06**	**4.46**
**Lingual gyrus**	**Left**	**-10**	**-66**	**-4**	**116**	**5.04**	**4.45**
Calcarine sulcus	Left	-14	-70	2		3.80	3.51
**Rolandic operculum**	**Right**	**58**	**14**	**4**	**60**	**4.64**	**4.16**
**Precentral gyrus**	**Left**	**-50**	**6**	**14**	**50**	**4.60**	**4.14**
**Supramarginal gyrus**	**Left**	**-52**	**-52**	**48**	**108**	**4.51**	**4.07**
-	Left	-56	-48	42		4.20	3.83
**Rolandic operculum**	**Left**	**-58**	**8**	**4**	**67**	**4.31**	**3.91**
**Lingual gyrus**	**Right**	**12**	**-64**	**-2**	**31**	**3.83**	**3.54**

Visual observation of roughness intensity ‘12’ – Resting

Regions	Side	MNI coordinates			Voxels	*T*	*Z*
		x	y	z			
**Inferior parietal lobule**	**Left**	**-44**	**-50**	**48**	**966**	**5.73**	**4.93**
-	Left	-36	-54	40		5.37	4.68
**Lingual gyrus**	**Left**	**-14**	**-76**	**-14**	**74**	**5.01**	**4.43**
**Inferior frontal gyrus**	**Left**	**-44**	**44**	**10**	**153**	**4.96**	**4.40**
-	Left	-34	46	16		4.89	4.35
**Inferior parietal lobule**	**Right**	**34**	**-56**	**38**	**138**	**4.93**	**4.38**
-	Right	42	-60	52		4.64	4.17
**Lingual gyrus**	**Right**	**20**	**-86**	**-18**	**160**	**4.81**	**4.29**
**Calcarine sulcus**	**Right**	**2**	**-94**	**-4**	**50**	**4.81**	**4.29**
**Inferior parietal lobule**	**Right**	**54**	**-44**	**46**	**56**	**4.52**	**4.07**
-	Right	50	-46	54		3.67	3.41
**Supplementary motor area**	**Left**	**-2**	**16**	**44**	**74**	**4.44**	**4.01**
-	Left	-2	20	54		4.20	3.83

Visual observation of roughness intensity ‘40’ – Resting

Regions	Side	MNI coordinates			Voxels	*T*	*Z*
		x	y	z			
**Superior occipital gyrus**	**Left**	**-10**	**-76**	**34**	**168**	**5.13**	**4.52**
**Inferior parietal lobule**	**Left**	**-42**	**-60**	**48**	**465**	**5.08**	**4.48**
-	Left	-50	-54	48		4.87	4.33
**Rolandic operculum**	**Left**	**-52**	**4**	**-2**	**86**	**5.04**	**4.45**
**Lingual gyrus**	**Right**	**8**	**-66**	**0**	**69**	**4.67**	**4.19**
**Precuneus**	**Right**	**8**	**-74**	**44**	**53**	**4.38**	**3.97**
**Postcentral gyrus**	**Right**	**22**	**-36**	**70**	**50**	**4.31**	**3.92**
**Lingual gyrus**	**Left**	**-10**	**-66**	**-4**	**46**	**4.23**	**3.86**
Calcarine sulcus	Left	-10	-64	4		4.20	3.83
**Insula**	**Right**	**52**	**12**	**-6**	**32**	**4.11**	**3.76**
-	Right	46	8	-2		3.92	3.61
**Supplementary motor area**	**Left**	**0**	**14**	**46**	**38**	**4.11**	**3.76**
**Angular gyrus**	**Right**	**40**	**-58**	**32**	**37**	**4.10**	**3.75**
**Insula**	**Left**	**-38**	**12**	**2**	**32**	**4.03**	**3.70**
-	Left	-34	12	10		3.88	3.57

Visual observation of roughness intensity ‘60’ – Resting

Regions	Side	MNI coordinates			Voxels	*T*	*Z*
		x	y	z			
**Inferior parietal lobule**	**Left**	**-36**	**-54**	**40**	**847**	**6.69**	**5.53**
-	Left	-46	-46	40		6.66	5.51
**Supplementary motor area**	**Left**	**0**	**16**	**56**	**130**	**5.53**	**4.79**
-	Left	-2	12	46		3.63	3.38
**Inferior frontal gyrus**	**Left**	**-38**	**40**	**8**	**124**	**5.42**	**4.72**
**Angular gyrus**	**Right**	**36**	**-58**	**38**	**205**	**5.15**	**4.53**
-	Right	38	-66	44		4.06	3.72
**Superior occipital gyrus**	**Left**	**-6**	**-102**	**8**	**53**	**5.00**	**4.43**
**Precentral gyrus**	**Left**	**-52**	**8**	**18**	**203**	**4.97**	**4.41**
Rolandic operculum	Left	-52	12	-2		4.87	4.33
**Calcarine sulcus**	**Right**	**20**	**-90**	**4**	**43**	**4.64**	**4.17**
**Inferior parietal lobule**	**Right**	**50**	**-48**	**46**	**71**	**4.54**	**4.09**
-	Right	48	-48	56		3.50	3.27
**Middle occipital gyrus**	**Right**	**28**	**-94**	**8**	**40**	**4.37**	**3.96**
-	Right	30	-92	16		3.87	3.57
**Inferior parietal lobule**	**Right**	**38**	**-58**	**58**	**32**	**4.11**	**3.76**
Angular gyrus	Right	44	-60	52		3.44	3.22
**Calcarine sulcus**	**Right**	**8**	**-84**	**10**	**40**	**3.87**	**3.57**
-	Left	-2	-84	14		3.49	3.26

Visual observation of roughness intensity ‘100’ – Resting

Regions	Side	MNI coordinates			Voxels	*T*	*Z*
		x	y	z			
**Superior temporal pole**	**Right**	**54**	**14**	**-6**	**418**	**6.22**	**5.24**
Insula	Right	38	8	2		5.75	4.94
**Middle occipital gyrus**	**Left**	**-52**	**-74**	**10**	**153**	**6.02**	**5.11**
Middle temporal gyrus	Left	-54	-68	2		5.56	4.81
**Rolandic operculum**	**Left**	**-52**	**6**	**-4**	**750**	**5.73**	**4.93**
-	Left	-54	-68	2		5.27	4.62
**Calcarine sulcus**	**Right**	**28**	**-94**	**6**	**337**	**5.63**	**4.86**
Lingual gyrus	Right	20	-84	-2		5.03	4.45
**Lingual gyrus**	**Left**	**-20**	**-82**	**-20**	**338**	**5.57**	**4.82**
-	Left	-10	-64	2		5.29	4.63
**Supramarginal gyrus**	**Right**	**66**	**-36**	**36**	**267**	**5.28**	**4.62**
-	Right	64	-44	36		4.58	4.12
**Calcarine sulcus**	**Left**	**-12**	**-102**	**8**	**118**	**5.22**	**4.58**
-	Left	0	-98	2		4.67	4.19
**Supramarginal gyrus**	**Left**	**-58**	**-46**	**38**	**631**	**4.89**	**4.34**
-	Left	-60	-26	42		4.87	4.33
**Middle frontal gyrus**	**Left**	**-42**	**42**	**26**	**49**	**4.32**	**3.93**
-	Left	-32	44	34		3.85	3.56
**Calcarine sulcus**	**Right**	**2**	**-88**	**14**	**46**	**4.11**	**3.76**
**Supplementary motor area**	**Right**	**6**	**0**	**68**	**49**	**4.03**	**3.70**
-	Right	2	10	64		3.62	3.37

Supplementary Table S6. Classification accuracies derived from the two folds of cross-modal decoding analysis.

	ACC	INS (left)	INS (right)	SMG
Tactile->Visual	26.8 %	25.2 %	26.9 %	25.9 %
Visual->Tactile	28.4 %	26.0 %	25.8 %	25.9 %

Supplementary Table S7-S9. Summary of correlation analysis for each identified cluster using single subject data. * indicates data showing significant correlation.

<u>Correlation results of identified brain regions from the searchlight analysis of tactile explorations</u>

Participant number	ACC		SMG	
	correlation coefficient (r)	p-value	correlation coefficient (r)	p-value
P01	0.18	0.28	0.52	0.03*
P02	-0.48	0.85	0.17	0.45
P03	-0.77	0.94	0.49	0.04*
P04	0.36	0.16	0.13	0.35
P05	0.10	0.36	0.46	0.05*
P06	-0.14	0.73	0.12	0.41
P07	-0.25	0.71	-0.21	0.70
P08	0.47	0.09	0.05	0.42
P09	-0.24	0.69	0.50	0.03*
P10	0.04	0.47	0.65	0.01*
P11	-0.22	0.57	-0.09	0.54
P12	-0.38	0.77	0.48	0.04*
P13	0.26	0.26	-0.12	0.60
P14	-0.33	0.80	-0.20	0.74
P15	-0.18	0.73	0.58	0.03*

Correlation results of identified brain regions from the searchlight analysis of visual observations

Participant number	PPC (left)		PPC (right)		S1		V1	
	correlation coefficient (r)	p-value	correlation coefficient (r)	p-value	correlation coefficient (r)	p-value	correlation coefficient (r)	p-value
P01	-0.24	0.74	-0.24	0.74	0.17	0.35	0.59	0.04*
P02	-0.03	0.49	-0.03	0.49	-0.44	0.91	-0.34	0.76
P03	-0.21	0.64	-0.21	0.64	-0.19	0.70	-0.07	0.63
P04	-0.08	0.61	-0.08	0.61	-0.28	0.83	-0.18	0.71
P05	0.15	0.27	0.15	0.27	-0.04	0.50	-0.16	0.55
P06	0.16	0.31	0.16	0.31	0.39	0.22	-0.16	0.71
P07	0.04	0.55	0.04	0.55	0.08	0.37	-0.02	0.51
P08	-0.63	0.96	-0.63	0.96	0.41	0.17	0.45	0.14
P09	-0.18	0.70	-0.18	0.70	0.06	0.41	-0.43	0.89
P10	0.55	0.05*	0.55	0.05	-0.52	0.87	0.35	0.20
P11	0.01	0.47	0.01	0.47	0.11	0.34	-0.04	0.60
P12	-0.13	0.64	-0.13	0.64	0.01	0.47	-0.38	0.85
P13	0.44	0.17	0.44	0.17	0.59	0.05*	-0.18	0.66
P14	-0.31	0.80	-0.31	0.80	-0.17	0.70	-0.15	0.64
P15	-0.38	0.88	-0.38	0.88	-0.34	0.75	-0.53	0.84

Correlation results of identified brain regions from the searchlight analysis of cross-modal decoding

Participant number	ACC		SMG		INS (left)		INS (right)	
	correlation coefficient (r)	p-value	correlation coefficient (r)	p-value	correlation coefficient (r)	p-value	correlation coefficient (r)	p-value
P01	0.27	0.23	0.56	0.04*	0.02	0.43	-0.19	0.63
P02	-0.27	0.81	-0.20	0.59	-0.23	0.68	-0.13	0.64
P03	0.01	0.36	-0.24	0.80	0.39	0.26	-0.20	0.63
P04	0.05	0.53	-0.68	0.95	-0.07	0.64	0.55	0.03*
P05	-0.12	0.65	0.47	0.05*	-0.44	0.84	-0.93	0.99
P06	0.66	0.03*	0.55	0.02*	-0.75	0.95	-0.43	0.82
P07	0.21	0.29	0.41	0.06	-0.63	0.97	0.26	0.19
P08	0.45	0.05*	-0.20	0.61	0.13	0.36	-0.27	0.77
P09	0.03	0.49	0.26	0.28	0.22	0.28	0.34	0.17
P10	0.56	0.12	0.49	0.05*	0.63	0.03*	0.23	0.23
P11	-0.16	0.63	0.13	0.38	0.49	0.15	0.10	0.43
P12	0.16	0.31	0.59	0.01*	-0.04	0.53	0.72	0.03*
P13	0.22	0.29	0.01	0.55	-0.01	0.56	0.02	0.55
P14	-0.30	0.74	0.50	0.05*	-0.02	0.55	0.01	0.38
P15	-0.40	0.89	-0.60	0.95	-0.66	0.97	-0.72	0.96
